# FACS-Based Graph Features for Real-Time Micro-Expression Recognition

**DOI:** 10.3390/jimaging6120130

**Published:** 2020-11-30

**Authors:** Adamu Muhammad Buhari, Chee-Pun Ooi, Vishnu Monn Baskaran, Raphaël C. W. Phan, KokSheik Wong, Wooi-Haw Tan

**Affiliations:** 1 Faculty of Engineering, Multimedia University, Persiaran Multimedia, Cyberjaya 63100, Selangor, Malaysia; cpooi@mmu.edu.my (C.-P.O.); twhaw@mmu.edu.my (W.-H.T.); 2 School of Information Technology, Monash University Malaysia, Subang Jaya 47500, Selangor, Malaysia; vishnu.monn@monash.edu (V.M.B.); raphael.phan@monash.edu (R.C.W.P.); wong.koksheik@monash.edu (K.W.)

**Keywords:** facial expression, micro-expression, emotion recognition, real-time classification, feature extraction

## Abstract

Several studies on micro-expression recognition have contributed mainly to accuracy improvement. However, the computational complexity receives lesser attention comparatively and therefore increases the cost of micro-expression recognition for real-time application. In addition, majority of the existing approaches required at least two frames (i.e., onset and apex frames) to compute features of every sample. This paper puts forward new facial graph features based on 68-point landmarks using Facial Action Coding System (FACS). The proposed feature extraction technique (FACS-based graph features) utilizes facial landmark points to compute graph for different Action Units (AUs), where the measured distance and gradient of every segment within an AU graph is presented as feature. Moreover, the proposed technique processes ME recognition based on single input frame sample. Results indicate that the proposed FACS-baed graph features achieve up to 87.33% of recognition accuracy with F1-score of 0.87 using leave one subject out cross-validation on SAMM datasets. Besides, the proposed technique computes features at the speed of 2 ms per sample on Xeon Processor E5-2650 machine.

## 1. Introduction

Micro-expression (ME) is described as a brief facial expression which appears on a person’s face according to the emotions being observed. ME occurs when people deliberately try to conceal their emotions, or unconsciously repress their emotions [1]. ME becomes more likely when there is more risk of revealing the emotions in a high-stake environment.

ME contains significant amount of information about the actual emotions of a person. These emotions maybe useful for applications including healthcare, security and interrogations [2]. However, extracting this information is highly challenging due to the subtleness of facial muscles movements in ME. This is mainly because the features are needed to be more descriptive. Moreover, another challenge is the duration ranging from 1/25 to 1/5 of a second, which is one of the main characteristics of ME [1].

In spite of these constrain, ME continues to attract the attention of researchers in the computer vision domain due to its vast potentials in security and interrogations, healthcare, and automatic recognition for real-time applications. In fact, current state-of-the-art methods are able to spot micro-level emotions with accuracies ranging between 65% and 80%. This in turn increases the viability of current ME techniques for real-world implementation. However, for ME system to perform in a real-time system, the following challenges need to be addressed:

1. Reliability of accuracy—A real-time ME system needs to be able to reliably spot micro-emotions from a person face. Existing ME techniques are however limited to certain datasets which curtails its reliability in multifarious settings.
2. Computational performance—Given that MEs usually last for a very short duration, it is imperative for a ME system to be able to process and classify a person’s emotion in real-time. Although existing approaches in ME recognition emphasizes on accuracy, the computational complexities of these approaches are not readily applicable for a real-time system.
3. Automatic onset-offset frames detection—Current state-of-the-art approaches in ME with high accuracies actually requires pre-defined spotting of the onset and offset frames. These constrain are not viable in real-time environment whereby the onset or offset frames cannot be pre-determined.

Generally, the process of recognising micro facial expression is divided into three parts, namely pre-processing, feature extraction and classification. Each part here plays an important role towards reliably classifying a person’s emotion. However, for automatic ME recognition, the features extracted should be more descriptive due to the subtleness of facial movement. Currently, the common feature extraction methods used for automatic ME recognition are Local Binary Pattern histogram from Three Orthogonal Planes (LBP-TOP) [3], Histogram of Oriented Gradients (HOG) [4] and Histograms of Oriented Optical Flow (HOOF) [5].

LBP-TOP represents a popular feature extraction method which considers the co-occurrences statistics in three directions (i.e., XY, XT and YT) of a video sequence. Here, *X*, *Y* and *T* represent the width, height and number of frames in a video stream, respectively [6]. However, the $O(n^{3})$ time complexity of LBP-TOP renders it computationally expensive as a real-time application. Attempts were made to accelerate the performance of LBP-TOP for ME recognition [7,8] with GPU computing platform. However, these methods recorded lower accuracies (i.e., 50%) and lacks clear indication on frame rate.

For the HOG feature extraction approach, the number of occurrences of gradient orientation in localized portions of an image (e.g., detection window, region of interest) is counted. The study in Reference [9] implemented 3D gradient histogram descriptor (HOG 3D) that computes features at the speed of 3.3 ms per sample. However, this method manually selects relevant regions based on Facial Action Coding System (FACS) [10] movement so that unwanted regions of the face are removed. Another study in Reference [11] proposed a FACS based method that utilizes a template of 26 defined facial regions. This method applies 3D HOG to extract temporal features of each region, and then utilizes Chi-square distance to find subtle facial motion in the local regions. However, the approaches presented in References [9,11] of defining movement within the selected regions are computationally expensive and therefore not suitable for real-time application. Though, study in Reference [12] attempted to improve the computation performance of HOG, but it was not tested for ME recognition.

On the other hand, Reference [13] proposed a Bi-Weighted Oriented Optical Flow (BI-WOOF) feature descriptor that implements local and global weight of HOOF descriptor. The reported results in Reference [13] demonstrates promising performance of ME recognition using only the onset-frame and the apex-frame in order to reduce the computational time. While Reference [14] proposed a feature descriptor that are less sensitive to the change in pose, illumination, and so forth, to increase the reliability of ME recognition for practical application. Another study in Reference [15] proposed an optical flow features from Apex frame Network to compute the optical strain features. Using a multi-database (i.e., SMIC, CASMEII and SAMM) setup with leave-one-subject-out cross-validation experimental protocol, these methods achieve ME recognition as high as 74.60%.

Although the aforementioned methods demonstrate notable improvements in ME accuracy, the high computational cost and requirements for pre-defined spotting of onset and offset frames renders these methods impractical as a real-time solution. Looking into macro-expression detection and recognition, References [16,17,18] suggested that geometric features are more robust in spotting the changes in face components, in comparison to the appearance based features using LPB-TOP, HOG and HOOF. However, to the best of our knowledge, very few articles utilize the geometric features for ME recognition based on single-frame sample. Existing geometric-based feature extraction algorithms yield poor ME recognition accuracy. This is due to the fact that geometric approach require large number of features [19]. However, since some of the existing ME datesets are FACS-coded. This suggests that the geometric features based on FACS could improve the recognition accuracy challenges. Therefore, this paper puts forward a geometric-based feature extraction technique using FACS for ME recognition with facial landmarks. The proposed method here addresses both the accuracy and computational cost for real-time ME. Crucially, the proposed technique processes ME recognition on frame-based samples, which substantially increases its feasibility in processing video of high frame rates. It computes features using facial landmarks extracted from the pre-processing stage of any input frame. This in turn substantially reduces the computational complexity in processing high frame rate video while at the same time improves the ME recognition accuracy further in comparison to the latest published article using the same validation technique [15].

The main contributions of this paper are:

1. FACS-based graph features using facial landmarks is proposed for real-time ME recognition. The proposed technique addresses both the accuracy and computational cost for real-time ME systems. The proposed technique computes features for ME recognition based on single-frame sample only, which substantially increases its feasibility of ME recognition with high speed camera.
2. Implementation of large-sample validation technique for single-frame geometric based features. Thus, multiple frames were selected from each video sequence and represented as samples of every corresponding class, which in turn increases the total number of samples of every class per dataset.

The rest of the paper is organized as follows: Section 2 reviews the related work. Section 3 formulates the proposed feature extraction algorithm based on FACS graph with facial landmark points, and Section 4 describes the dataset restructuring for frame-based sample analysis. Section 5 presents the experimental results and analyzes the performance for different spontaneous dataset and concludes this paper.

## 2. Related Work

Comprehensive review on automatic ME recognition and analysis challenges have recently been presented in Reference [20], focusing on the clarification on how far the field has come, identifying new goals, and providing the results of the baseline algorithms. As reported in Reference [20], feature extraction improvement is the main focus in the existing studies of ME detection and recognition. Studies in References [3,21,22] suggest accuracy improvement is more significant by employing an additional pre-processing to enhance quality of data before feature extraction process [23]. However, implementation of the existing pre-processing approaches, such as TIM [3], emotion magnification [21], and filtering [22], introduces more computational cost challenges. Besides, to the best of our knowledge, there is no published article until date towards real-time implementation of these pre-processing methods for automatic ME spotting.

Hence, an acceleration of feature extraction has become necessary for real-time ME recognition in order to attain high throughput. In addition, from the feature perspective for ME recognition, there are three major approaches, namely—appearance-based approach, dynamic approach and geometry-based approach. Based on reported results in Reference [24], both appearance-based and dynamic approaches are not feasible for real-time systems on low-level systems as they involve high cost computations. However, Reference [7] proposed an appearance-based feature extraction method described as fast LBP-TOP using the concept of tensor unfolding to accelerate the implementation process from 3D-space to 2D-space. This method improves the computational time by 31.19 times on average when compared to the original LBP-TOP implemented in Reference [7]. Moreover, Reference [8] proposed another appearance-based feature extraction method by computing conventional LBP-TOP using many-core graphics processing unit (GPU) with CUDA parallel computing platform [8]. The proposed algorithm in Reference [8] increases the performance speedup up to 130× faster against the serial algorithm, with 1120 × 1360 video resolution. However, References [7,8] neither measure nor present the frame rate of their accelerated LBP-TOP algorithms, which make no conclusions for the feasibility of computing in real-time automatic ME recognition. Thus, in fairness conclusions of computational complexity as suggested by Reference [17,18], geometric-based approach is the best option towards realization of real-time ME recognition system as it involves low complexity computations of facial muscle movement. In addition, there is no requirements of onset-offset detection for geometric-based approach, which substantially increases its feasibility in processing video of high frame rate.

Geometry-based feature extraction approach deals with symmetrical features that gives the locations and shapes of facial components [25]. The study in Reference [26] presented graph-based features that locate and define points into regions of face in order to compute features, and then recognition of emotions is done by using corresponding feature vector. Moreover, Reference [27] proposed a new face expression recognition method based on extracting discriminative features. The study in Reference [14], the proposed method utilizes local statistical features from a region-of-interest and applied AU codes to detect ME. Action Units (AU) are the fundamental actions of individual muscles or groups of muscles, and FACS involves 44 AUs related to visually discernible facial muscle activation. Moreover, FACS defines AU intensities on a five-point ordinal scale (i.e., from lowest *A* to strongest *E* intensity. The main benefit of estimating AU strengths is that the qualified AUs would yield more information about the emotional involvement of a subject. Moreover, since humans can express their feelings in different ways under different situations, information conveyed by AU intensities can be exploited to adapt emotion recognition. Table 1 summarizes the advantages and disadvantages of the aforementioned feature extraction approaches.

To date, the achievement of ME recognition accuracy using spontaneous ME datasets ranges from 40% to 88% using different validation approaches including leave one subject out cross validation, leave one video out cross validation and k-fold cross validation. For methods tested using all classes, the highest accuracy is 88.28% with F1-score of 0.87 with OFF-ApexNet method from Reference [15] over CASMEII dataset. As reported in Reference [28], the uneven distribution samples among classes create more challenges that impacts recognition rate. The trend of ME recognition is also changing from low-level hand-crafted feature to high-level approaches. However, the development of high-level approach is restricted by small dataset sizes. Hence, augmentation of data or transfer learning is done to provide higher number of samples. The study in Reference [29] present deep learning model named spatio-temporal recurrent convolutional networks (STRCN), and the reported ME recognition accuracy is 80.3% with F1-score rate of 0.75 on CASMEII dataset. Moreover, another study in Reference [30] presents a shallow triple stream 3D CNN (STSTNet) that is computationally light whilst capable of extracting discriminative high level features and details of MEs. The reported results active up to 76.05% of recognition accuracy with 0.74 F1-score rate on a combined dataset created from SMIC, CASMEII and SAMM datasets.

While the aforementioned studies lay a solid groundwork in ME recognition accuracy, the computation performance based on speed per frame remains unreported. Moreover, with the current advancement of technology for real-time machine learning based systems for automatic ME recognition, it is necessary to have a reliable feature extraction algorithm for real-time implementation of ME recognition systems. Looking into the FACS-based features, where a trained coder views facial geometric movements and expressions in video sequences, and then observe each muscle movements as AU. FACS is described as efficient, objective and comprehensive technique to present facial expression without any downside [31], and it is widely accepted by many researchers in the field of psychology and physics. FACS devised 46 AUs, where the expressions to represent human emotional states are produced by the movements of AUs or their combination based on these system. Thus, identifying AUs based on facial muscles movement for ME recognition could address the computational challenges for real-time application. In this regard, this paper puts forward a FACS-based graph features using facial landmarks for real-time ME recognition systems. Crucially, the proposed feature extraction algorithm improves the recognition accuracy as well as the computation complexity. The following section presents the formulation and implementation of the proposed algorithm.

## 3. Proposed Algorithm

**Figure 1:**
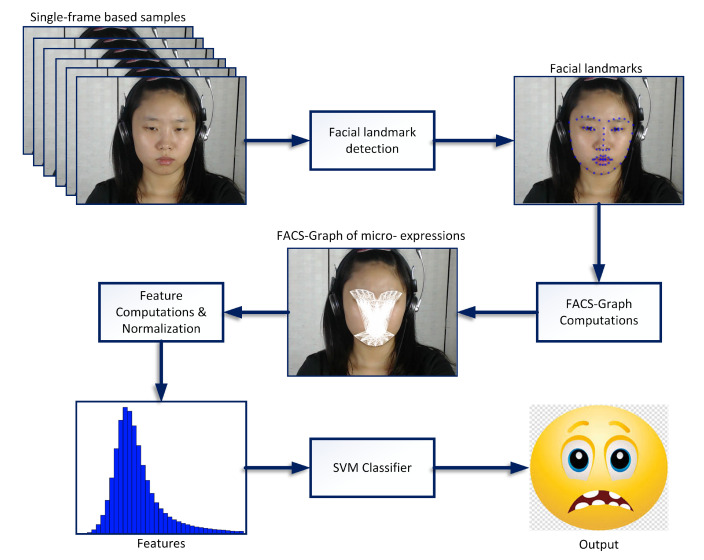
Principle of the proposed Facial Action Coding System (FACS)-based graph features for Micro-expression (ME) recognition.

This section presents the proposed facial feature extraction algorithms for ME recognition. Figure 1 shows the flow of processes for real-time ME detection and classification using the proposed feature extraction.

The real-time system utilizes a high speed camera to capture video frames, then Facial Detection algorithm is applied to identify the presence of face for micro-emotion analysis within the processing frame. For every successful detected face, 68 landmark points will be identified for the subject’s facial components. Subsequently, the proposed technique utilizes these landmark points to compute FACS-based graph for different emotions, and then the distance and gradient of segments from the computed graphs are presented as features. These features are normalized and then presented to the Classifier for ME detection and recognition.

The authors of Reference [32] demonstrated that the geometric variation of these features can be utilized to identify different facial emotions. However, the challenge of this technique is to correctly identify the effective region that represents each facial emotion for recognition. Thus, this paper presents a new method that utilizes facial landmark based graph to compute features. This paper analyse the geometric features using two methods, namely: (i) Full-face graph and (ii) the proposed FACS-based graph. For both methods, the dlib [33] facial landmark detection tool were utilized for facial landmark detection. This tool utilizes histogram of oriented gradients (HOG) face detector to provide a 68-point model that represents a face shape, eyebrow, eyes, nose and mouth. The dlib facial landmark detection is able to achieve high speed performance and accurate in comparison to other facial detection methods [34].

Algorithm 1 presents the first method, that is, feature computation using the Full-face facial graph. This algorithm computes a total of 2278 one-to-all segments generated from the 68-points facial landmarks for every sample (i.e., single frame samples). Here, $L_{n}$ represents the facial landmark points as input data, where *n* represents the index for the *x* and *y* coordinates of a landmark point. Then, $F_{k}$ represents the computed features as output data (i.e., the results), where *k* represents the number of computed elements. As shown in Algorithm 1, two feature elements are computed from every segment, where the first element is the distance between the two points computed using Euclidean algorithm and the second element is the gradient of the two points computed using slope equation. Thus, the total number of feature elements computed from the 2278 segments is 4556.
**Algorithm 1:** Feature computation with full-face graph.
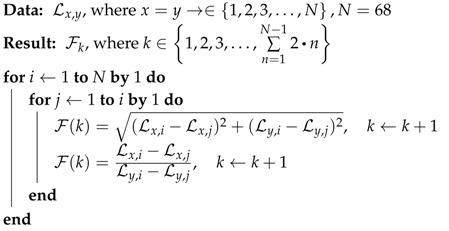


On the other hand, Equations (1)–(3) express the FACS-based graph computation using the facial landmarks. Firstly, Equation (1) groups the AU codes based on FACS by computing the landmark coordinates of every connecting points of facial components defined within the AU region, where $\sigma_{p}$ represent the first connecting point and $\sigma_{q}$ represent the second connecting point. Then, Equation (2) combines the AUs codes defined from Equation (1) to generate graphs for every emotion (denoted as δ), where *R* represents the number of AUs for per emotion. While, Equation (3) groups the generated graphs of all the seven emotions computed using Equation (2) to form the combined graph (denoted as λ), where *K* represents the total number of emotions considered in this work. Equation (4) deletes the repeated segments within the combined graph (i.e., λ) in order to produce the final FACS-based graph (denoted as ζ). Total number of segments computed from Equation (3) is 3083. Then, after removing the repeated segments using Equation (4), the new total number of segments is reduced to 1178.

(1) $$\mathrm{AU}=\left\{\sigma_{p},\sigma_{q}\right\},\;p\in \left\{1,2,3,\ldots P\right\}\;and\;q\in \left\{1,2,3,\ldots p\right\}$$

(2) $$\delta =\left\{{\mathrm{AU}}_{1},{\mathrm{AU}}_{2},{\mathrm{AU}}_{3},\ldots {\mathrm{AU}}_{R}\right\}$$

(3) $$\lambda =\left\{\delta_{1},\delta_{2},\delta_{3},\ldots \delta_{K}\right\},\;\to K=7.$$

(4) ζ=unique(λ)

Algorithm 2 describes how the features are computed using the FACS-based graph. Similarly, Algorithm 2 computes two feature elements for every segment, and this process is repeated for all the segments of the FACS-based graph to compute the complete 2356 (i.e., 1178 × 2) features.
**Algorithm 2:** Feature computation with FACS-based graph.
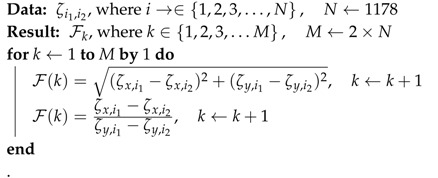


As observed here, the total features computed with Algorithm 2 are lesser in comparison with the features computed in Algorithm 1. Note that the features are computed in the same manner in methods after the graph formation using Equation (1)–(4) (as described in Figure 1). To further elaborate the proposed FACS-based graph features, Table 2 lists the facial region grouping for AUs defined based on landmarks using FACS codes.

As shown Table 2, the first column lists the emotions classes (denoted as δ), second column lists the number of AUs for each emotion, while the third column lists the AU codes based on FACS, and then the fourth column lists the grouping of facial components landmarks per AU. Here, the 68 points facial landmarks is divided into seven facial units namely; lower-jaw, left-eyebrow, left-eye, right-eyebrow, right-eye, nose and mouth, which are defined as LJ, LEB, LE, REB, RE, N and M respectively. Furthermore, the grouping of facial components landmarks consist of one part for AU∈{12,14,15,16,23,26} and two parts for AU∈{1,2,4,5,6,7,9,20}. For the grouping with one part, the sets of landmarks from the facial components within the AU region are combined to form a single set, then using Equation (1), the segments are computed to form a graph of AU. While, for grouping with two parts, segments are computed in similar way as described for grouping with one part, and then the two groups of segments are combined (shown in Table 2, column four using ∪) to form a graph of AU.

Table 3 tabulates the list of AUs and descriptions for facial muscle movement according to Reference [10]. As shown in Table 3, the first column lists the class of emotions, while the second column lists the marked-samples with arrows showing the direction of face muscle movement for each class of emotion, then the third column lists the combination of AUs to represent each class of emotion, and the last column lists the FACS name for all AUs for the corresponding class of emotions.

**Figure 2:**
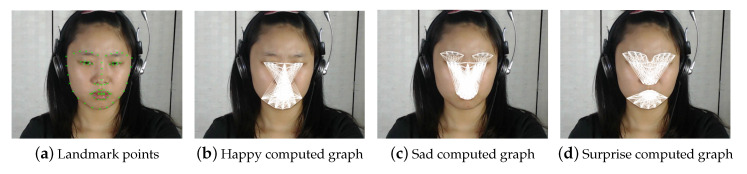

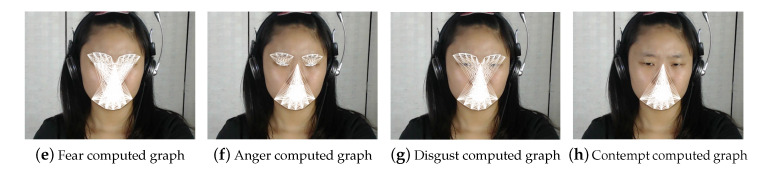
FACS-based graphs of different emotions.

**Figure 3:**
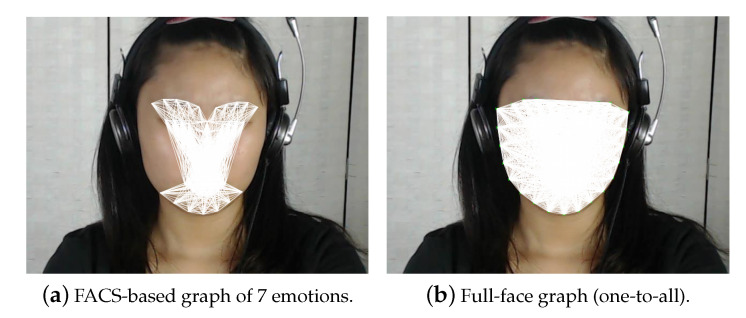
FACS-based graph vs Full-face graph.

To further describe the FACS-based graphs for each emotion, Figure 2 present a sample image (mid-frame of subject 1 from CAS(ME)${}^{2}$ dataset), where Figure 2a maps the 68 landmarks on the targeted facial components. While, Figure 2b–h maps the proposed FACS-based graph generated for different emotions. For further illustration, Figure 3 compares Algorithm 1 and the proposed FACS-based graph. Specifically, Figure 3a shows the FACS-based graph that combines all the 7 emotions graphs (i.e., graphs in Figure 2b–h) into one graph of all the 7 emotions, while Figure 3b shows the Full-face method. Here, each segment indicates a process of distance and gradient computation. Therefore, this suggests that the FACS-based features (i.e., Figure 3a) have fewer computational processes compared to the Full-face features.

To further justify our motive of the proposed single frame-based analysis for fast computation and better accuracy, this paper analyzes the performance of the single frame-based approach under three different setups. For each setup, set(s) of samples are extracted using the corresponding Equation from (5) to (7). More details of these Equations are presented in Section 4.

(5) $$\chi =\frac{\left|\right|S^{\nu }\left|\right|}{2}$$

(6) $$S_{\mathrm{mid}-\mathrm{three}}^{\iota }=\left\{\chi -1,\chi ,\chi +1\right\}$$

(7) $$S_{\mathrm{mid}-\mathrm{half}}^{\iota }=\left\{S_{\left(\frac{\chi }{2}+1\right)}^{\nu },S_{\left(\frac{\chi }{2}+2\right)}^{\nu },\ldots S_{\left(\frac{\chi }{2}+\chi \right)}^{\nu }\right\}.$$

## 4. Experiment Setup

In this section, the experimental setups using four available spontaneous datasets are presented. These datasets are: Spontaneous Micro-Expression dataset (SMIC), Chinese Academy of Sciences Micro-Expression (CASMEII), Spontaneous Actions and Micro-Movements (SAMM) and A Dataset of Spontaneous Macro-Expressions and Micro-Expressions (CAS(ME)${}^{2}$).

### 4.1. SMIC

The SMIC dataset is spontaneous with 20 participants (6 females and 14 males) collected using high speed (HS) camera set to 100 fps with resolution of 640 × 480 and normal visual camera (VIS) and near-infrared (NIR) set to 25 fps with resolution of 640 × 480 as well [3]. The accepted duration of ME for SMIC is 500 ms. Since not every participant showed ME when recording, thus the final dataset includes total 164 ME clips from 16 participants recorded in HS dataset. This database contains three emotion classes: (1) negative (which presents: sad, fear, disgust), (2) positive (which presents: happiness) and (3) surprise.

### 4.2. CASMEII

CASMEII dataset is an improved version of CASME dataset [35]. CASMEII dataset includes both spontaneous and dynamic ME collected using a high speed camera with 200 fps with resolution of 280 × 240 [36]. This dataset contains total 247 ME from 35 participants selected from nearly 3000 facial movements and have been labeled with AUs based on FACS [37]. This database contains five emotion classes: (1) happiness, (2) disgust, (3) surprise, (4) repression and (5) others. SAMM dataset is the first high-resolution dataset of 159 spontaneous ME with largest variability in demographics [38].

### 4.3. SAMM

The SAMM dataset is collected using a high speed camera with 200 fps with resolution of 2040 × 1088. SAMM dataset was designed in such was that each video stimuli was tailored to each participant, rather than getting self-reports after the experiment. This allowed for particular videos to be chosen and shown to participants for optimal inducement potential [38]. This database contains seven emotion classes: (5) contempt, (2) disgust, (3) fear, (4) anger, (5) sadness, (6) happiness and (7) surprise. CAS(ME)${}^{2}$ dataset is the latest ME database with both macro-expression and ME. This database includes 250 macro-expression and 53 ME samples selected from more than 600 facial move-expression [39].

### 4.4. CAS(ME)${}^{2}$

CAS(ME)${}^{2}$ dataset is spontaneous with 22 participants (16 females and 6 males) collected using camera at a speed of 30 fps with resolution of 640 × 480. CAS(ME)${}^{2}$ has been labelled using combinations of AUs, self-reports and the emotion category decided for the emotion-evoking videos. This database contains four emotion classes: (1) positive, (2) negative, (3) surprise and (4) other.

The reasons of using these datasets for experiments are; first, the datasets are classified as spontaneous, which indicates that the emotions captured from the participants are genuine, then secondly, to compare the performance of the proposed feature extraction technique of FACS-coded datasets (i.e., CASMEII and SAMM) with non-FACS-coded datasets (i.e., SMIC and CAS(ME)${}^{2}$). Table 4 summarizes the selected spontaneous ME datasets used in this work. First column lists the video frame rate, second column lists image resolution, third column lists image duration of ME, fourth of column lists the number of participants, fifth column lists the number of samples, sixth column lists the emotions classes and the last column lists the FACS-coded samples.

As mentioned in Section 1, the main goal of this paper is to achieve fast automatic ME recognition for real-time application. Therefore, the proposed FACS-based graph feature extraction approach is based on single-frame sample. From the three setups of experiments, Equation (5) expresses the selection of only the middle-frame from each video sequence and represent as sample of that class of emotion for the first experimental setup, while Equation (6) expresses the selection of mid-three frames from each video sequence to represent sets of samples of that class of emotion for the second experimental setup, and Equation (7) expresses the selection of mid-half frames from each video sequence to represent sets of samples of that class of emotion for the third experimental setup.

### 4.5. Setup of Experiment I

**Figure 4:**
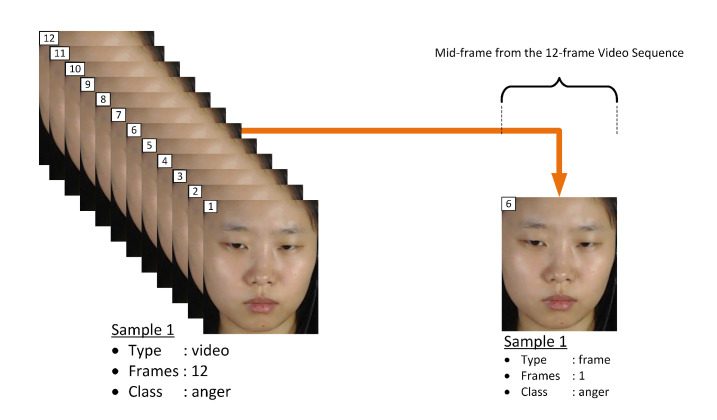
Illustration of video to frame sampling for Experiment I.

This experiment selects only the mid-frame and present it as the only input frame from each video, this process is expressed in Equation (5). As suggested in Reference [40], the mid-frame within each video sequence will have a significant change in comparison to the first (i.e., onset) and last (i.e., offset) frames, and therefore considered as the input frame to represent the video sample in this experimental setup. Figure 4 illustrates the selection of a sample from a 12-frame video sequence for Experiment I setup.

### 4.6. Setup of Experiment II

**Figure 5:**
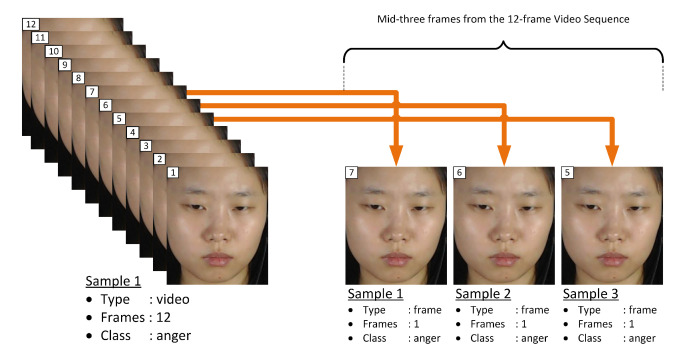
Illustration of video to frame sampling for Experiment II.

In this experiment, the frame-based datasets used for analysis are generated from mid-three frames of each video sequence using Equation (6). Figure 5 illustrates how Equation (6) computes three samples from a 12-frame video sequence for Experiment II setup.

### 4.7. Setup of Experiment III

**Figure 6:**
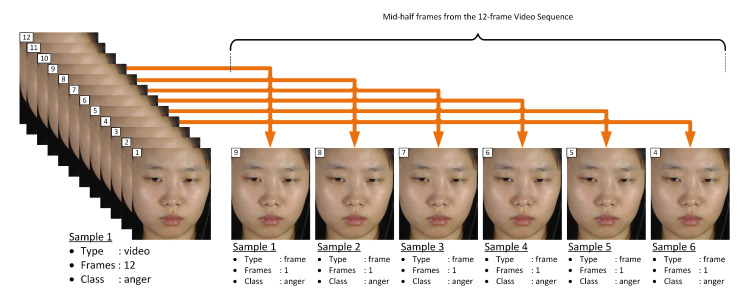
Illustration of video to frame sampling for Experiment III.

This experiment generates frame-based datasets with the mid-half frames from each video sequence using Equation (7). Figure 6 illustrates how Equation (7) generates 6 samples from a 12-frame video sequence for Experiment III setup.

The number of samples generated by using Equations (5)–(7) from the original video sequences for each experiment is recorded in Table 5. The measured results of these experimental setups are presented in Section 5. Support Vector Machine (SVM) is employed as the classifier.

## 5. Results and Discussion

To evaluate the proposed feature extraction algorithm tested on the single frame-based samples generated in this work, accuracy and F1-score are measured for four different datasets (i.e., SMIC, CASMEII, CAS(ME)${}^{2}$ and SAMM). Here, the accuracy refers to how good the predictions are on average, that is, “the number of emotion samples correctly predicted” by “the total number of testing samples”. On the other hand, the F1-score is the harmonic mean of precision and recall, where recall is the ratio of “the total amount of positive instances that were actually predicted”, while precision is the ratio of “positive instances among the predicted instances”. In addition, the validation technique used is leave-one-subject out cross validation (LOSOCV) in order to fit well with the frame-based samples.

Table 6 tabulates the results in terms of accuracy and F1-score. From these tables, Exp. I refers to the evaluation of the proposed algorithm with only the the middle frame from each video sequence to create single frame-based samples. While, Exp. II and III refers to the evaluation for mid-three and mid-half frames from the video sequence, respectively. From each experiment, features by Full-face graph and the proposed FACS-based graph are analyzed.

As shown in Table 6, Exp. I, with Full-face graph features, SMIC yields the lowest accuracy and F1-score (i.e., 63.54% and 0.58) and SAMM yields the highest accuracy and F1-score (i.e., 80.28% and 0.80). On the other hand, for FACS-based graph features, SMIC also yields the lowest accuracy and F1-score (i.e., 70.25% and 0.69), and SAMM yields the highest accuracy and F1-score (i.e., 87.33% and 0.87). In the case of Experiment II which considers Full-face graph features, SMIC yields the lowest accuracy and F1-score (i.e., 66.90% and 0.65) while SAMM yields the highest accuracy and F1-score (i.e., 74.00% and 0.70). Similarly, for FACS-based graph features, SMIC yields the lowest accuracy and F1-score (i.e., 76.67% and 0.75), while SAMM yields the highest accuracy and F1-score (85.85% and 0.84). Finally, for the case of Experiment III which considers Full-face graph features, also SMIC yields the lowest accuracy and F1-score (i.e., 62.34% and 0.60), while SAMM yields the highest accuracy and F1-score (i.e., 78.40% and 0.75). Similarly, for FACS-based graph features, SMIC yields the lowest accuracy and F1-score (i.e., 64.64% and 0.53) while SAMM yields the highest accuracy and F1-score (i.e., 81.43% and 0.81).

From these results, Experiment I outperformed Experiment II and III using the proposed FACS-based graph features analysis on SAMM with the highest accuracy and F1-score of 87.48% and 0.87, respectively. Similarly, Experiment I outperformed Experiment II and III using the Full-face graph features analysis on SAMM with the highest accuracy and F1-score of 80.28% and 0.80, respectively. As observed here, Experiment I achieved the highest accuracy due to two reasons; firstly, the size of samples per subject for each dataset is smaller and secondly, the selected frame (i.e., the presentation of mid-frame as the input frame from each video sequence) is more precise in comparison with other datasets (i.e., SMIC, CASMEII and CAS(ME)${}^{2}$).

To further evaluate the performance of the proposed graph algorithm, Table 7 and Table 8 record the accuracy and F1-score of the conventional methods considered for comparison against the proposed method. As shown in Table 7 and Table 8, studies from References [13,40,41,42,43], registered the highest accuracies of 64.02%, 62.90%, 68.29%, 54.88% and 54.00% with F1-score of 0.64, 0.62, 0.67, 0.53 and 0.52 over SMIC dataset. While, the studies in References [15,24,29] registered the highest accuracies of 76.60%, 80.30% and 88.28% with F1-score of 0.60, 0.75 and 0.87 over CASMEII dataset. On the other hand, the proposed algorithm with Full-face graph registered the highest accuracies of 66.54%, 73.45%, 74.41% and 80.28% with F1-score of 0.65, 0.70, 0.80 and 0.87 over SMIC, CASMEII, CAS(ME)${}^{2}$ and SAMM datasets, respectively. While the proposed algorithm with FACS-based graph registered the highest accuracies of 76.67%, 75.04%, 81.85% and 87.33% with F1-score of 0.75, 0.74, 0.80 and 0.87 over SMIC, CASMEII, CAS(ME)${}^{2}$ and SAMM datasets, respectively.

To sum up, the results presented in Table 7 and Table 8 suggest that the proposed FACS-based graph features outperformed the current state-of-the-art algorithms with accuracy and F1-score of 76.67% and 0.75 over SMIC, 81.85% and 0.80 over CAS(ME)${}^{2}$ and 87.33% and 0.87 over SAMM, respectively. However, the reported results from Reference [15] on CASMEII outperformed the proposed algorithm with accuracy and F1-score of 88.28% and 0.87, respectively. This suggests that the CASMEII datasets did not work well with the proposed algorithm, which could be due to the performance limitation of the landmarks detection tool used in our experiments (i.e., dlib tool).

In addition to the accuracy and F1-score, the computational time of the proposed feature extraction algorithm was investigated. The processing time of the proposed algorithm is analyzed on Xeon Processor E5-2650 v4 @ 2.4Ghz with 24 logical processors. The computation time taken to extract the features using one-to-all is approximately 3.1 ms. For the proposed feature extraction algorithm based on FACS, it takes approximately 2 ms to compute features per sample. Based on this analysis, the computational performance of the proposed feature extraction algorithm using either one-to-all or FACS-based significantly reduced the processing time of feature computation. This suggests that the proposed feature extraction algorithm is potential for real-time ME recognition with high speed camera integrated with fast facial landmark detection and accelerated SVM classification.

Table 9 lists the performances of computation time from References [7,8,44] towards the implementation of real-time ME recognition. Knowing that the reported processing time from each article was based on the machine used for analysis, and therefore, no conclusions on the processing time differences. As shown in Table 9, the first implementation of fast feature extraction algorithm by Reference [7] using tensor unfolding with GPU achieves up to 31.9× faster than the un-optimised LBP-TOP on CPU. The processing time for feature computation per sample with 50 × 50 is 107.39 ms. Similarly, Reference [8] implemented a GPU based LBP-TOP on CUDA programming platform and achieved an impressive performance of 2.98 ms for feature computation per sample with 140 × 170. On the other hand, Reference [44] proposed method that computes the difference of onset and apex-frames as features. As reported by the authors, this method achieves 9 ms of processing time per frame for 640 × 480.

While acknowledging the differences in computing resources may have potentially contributed to the superiority of our method in computation time, the benchmark algorithms require several pre-process stages including face detection from the original raw video sequence, face alignment, face cropping and onset-offset detection. These pre-processings introduce more challenges of computational time, which limits the performance of the accelerated feature extraction. In addition, these challenges have not been addressed by any research so far, which makes these algorithms more crucial for real-life applications.

On the other hand, in the proposed method, the computed features using all landmark points requires 3.1 ms. In addition, the computed features by using selected landmark points based on FACS requires 2 ms. On the contrary, based on the benchmark studies, the proposed algorithms requires facial landmark detection as the only pre-processing stage. Then, by using the facial landmark points obtained from the processing stage, as described in Section 3.

Thus, in comparison to the benchmark studies, the proposed FACS-based graph features achieve well above the required speed of 200 fps for real-time ME recognition, leaving 1.2 ms to compute the facial landmark detection and classification, while 0.8 ms to compute the face detection and classification for Full-face graph features. However, the performance of the proposed method is limited due to two major reasons, namely; (i) the definition of AUs are based on the FACS system presented in Reference [10], which is described as not perfect to give an objective stance and emotion mapping [24] and (ii) the instability of dlib facial landmark detection due to the factors including image lighting, subject pose and unintentional partial occlusion of subject face (such as wearing eyeglass or having long hair).

## 6. Conclusions

This paper presents the a fast feature extraction algorithm using facial landmark points for ME recognition in real-time. The proposed algorithm measures the geometric changes through the facial muscles to recognize micro emotions using 68 facial landmark points. The 68 landmark points are segmented based on all points for full face analysis or selected points based on FACS. This algorithm is analyzed using frame-based sample generated from the four spontaneous datasets using three different approaches (i.e., mid-frame, mid-three frames and mid-half frames). In the experiment, all generated frame-based samples from the video sequences are presented as as input frames for feature computation using the proposed algorithm. Results suggest that the best accuracy and F1-score achieved are 87.33% and 0.87 over the SAMM dataset using FACS-based graph features with only the mid-frame sampling approach. Furthermore, the proposed feature extraction algorithm based on FACS graph exhibits the best computational performance of 2 ms per sample. Therefore, this suggests that the presented feature extraction method outperformed the current state-of-the-art over SMIC, CAS(ME)${}^{2}$ and SAMM datasets. In addition, this method addresses the speed concern for real-time ME recognition requiring when integrated with fast facial landmark detection and accelerated SVM classifier. However, the accuracy still needs further improvements for real world applications.

### Future Work

The accuracy of the proposed method could be further improved by implementing geometric-based emotion magnification using the facial landmark points prior to computing the features. The magnification of subtle movements of facial components will enhance the visibility of micro-emotions and yield up to 90.0% and above of recognition accuracy, as reported in Reference [18] on macro-expression dataset. This is because, in general, geometric-based feature extraction methods works well with more distinct facial muscle movements in order to differentiate among emotions with similar features. 

## Figures and Tables

**Table 1:** Comparison between various feature extraction approaches.

Approaches	Implementation	Advantages	Disadvantages
Appearance-based	Pixel-wise level	- Small no of feature	- Need good quality image
			- Large database require
			- Illumination
Dynamic-based	Non-rigid motion changes	- Support two frames	- Need good quality image
			- High complexity
Geometry-based	Position of facial components	- Small database	- Require large no. of features
		- Low complexity	
		- Support single frame	

**Table 2:** Grouping of Action Unit (AU) facial regions of the proposed FACS-based graph.

*δ*	AUs	Codes	Grouping of Facial Regions	AU_*ℒ*_	AU_*𝒮*_
Happy	2	6	{LE,N}∪{RE,N}	30	435
		12	{N,M,LJ}	35	630
Anger	3	1, 4	{LEB,LE}∪{REB,RE}	22	110
		15	{N,M,LJ}	35	630
Sad	4	1, 2	{LEB,LE}∪{REB,RE}	22	110
		5	{LE}∪{RE}	12	30
		26	{M,LJ}	27	351
Fear	7	1, 2, 4	{LEB,LE}∪{REB,RE}	22	110
		5	{LE}∪{RE}	12	30
		7	{LEB,LE,N}∪{REB,RE,N}	40	380
		20	{LE,N,M}∪{RE,N,M}	70	1190
		26	{M,LJ}	27	351
Surprise	4	4	{LEB,LE}∪{REB,RE}	22	110
		5	{LE}∪{RE}	12	30
		7	{LEB,LE,N}∪{REB,RE,N}	40	380
		23	{M,LJ}	27	351
Disgust	3	9	{LEB,N}∪{REB,N}	28	182
		15	{N,M,LJ}	36	630
		16	{M,LJ}	27	351
Contempt	2	12,14	{N,M,LJ}	36	630

**Table 3:** AUs for Different Emotions.

Emotion	Sample Frame	Action Units	FACS Name
Happiness	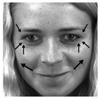	6 + 12	Cheek raiser Lip corner puller
Sadness	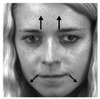	1 + 4 + 15	Inner brow raiser Brow lowerer Lip corner depressor
Surprise	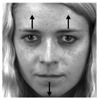	1 + 2 + 5B + 26	Inner brow raiser Outer brow raiser Slight Upper lid raiser Jaw drop
Fear	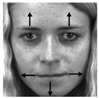	1 + 2 + 4 + 5 + 7 + 20 + 26	Inner brow raiser Outer brow raiser Brow lowerer Upper lid raiser Lid tightener Lip stretcher Jaw drop
Anger	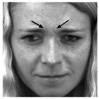	4 + 5 + 7 + 23	Brow lowerer Upper lid raiser Lid tightener Lip tightener
Disgust	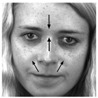	9 + 15 + 16	Nose wrinkler Lip corner depressor Lower lip depressor
Contempt	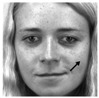	R12A + R14A	Lip corner puller (right side) Dimpler (right side)

**Table 4:** Spontaneous ME Datasets used for experiments.

Datasets	FRP	Image Resolution	Duration ( ms)	Sub.	Samp.	Class	FACS-Coded
SMIC [3]	100	640 × 480	500	20	164	positive	No
						negative	
						surprise	
CASMEII [36]	200	640 × 480	245	35	247	happiness	Yes
						surprise	
						disgust	
						repression	
						others	
SAMM [38]	200	2040 × 1088	500	32	159	happiness	Yes
						sadness	
						anger	
						surprise	
						fear	
						disgust	
						contempt	
CAS(ME)${}^{2}$ [39]	30	640 × 480	419	22	341	happiness	No
						anger	
						disgust	

**Table 5:** Number of Total Samples Extracted for Experiment I, II and III.

Datasets	γ	Samples			Classes
		Exp. I	Exp. II	Exp. III	
SMIC	20	164	492	1484	3
CASMEII	35	247	741	4352	5
CAS(ME)${}^{2}$	22	341	1023	3055	4
SAMM	32	159	477	2992	7

**Table 6:** Experimental Results (Accuracy & F1-score) for leave-one-subject out cross validation (LOSOCV).

Setup	Features	Accuracy (%)				F1-Score			
		SMIC	CASMEII	CAS(ME)${}^{2}$	SAMM	SMIC	CASMEII	CAS(ME)${}^{2}$	SAMM
Exp. I	Full-face	63.54	73.45	72.83	80.28	0.58	0.60	0.72	0.80
	FACS-based	70.25	75.04	81.41	87.33	0.69	0.74	0.79	0.87
Exp. II	Full-face	66.90	71.93	70.05	74.00	0.65	0.70	0.69	0.70
	FACS-based	76.67	74.07	81.85	85.04	0.75	0.72	0.80	0.84
Exp. III	Full-face	62.34	71.31	74.41	78.40	0.60	0.68	0.71	0.75
	FACS-based	64.64	64.87	74.62	81.43	0.53	0.67	0.72	0.81

**Table 7:** LOSOCV: Accuracy of the proposed algorithm vs other methods.

Papers	Features	Classifier	Accuracy (%)			
			SMIC	CASMEII	CAS(ME)${}^{2}$	SAMM
[13]	Bi-WOOF	SVM	62.20	58.85	59.26	-
[15]	OFF-ApexNet	CNN	67.68	88.28	-	68.18
[24]	LBP-TOP	SMO	-	68.24	-	54.93
	HOOF	SMO	-	76.60	-	60.06
	HOG3D	SMO	-	69.87	-	63.93
[29]	STRCN-A	deep-RCN	53.10	56.00	-	54.50
	STRCN-G	deep-RCN	72.30	80.30	-	78.60
[40]	Facial Dynamics Map	SVM	54.88	45.93	-	-
[41]	STCLQP	SVM	64.02	58.39	-	-
[42]	Bi-WOOF + Phase	SVM	68.29	62.55	-	-
[43]	Hierarchical STLBP-IP	KGSL	54.00	46.00	-	-
Proposed	Full-face graph	SVM	66.54	73.45	74.41	80.28
	FACS-based graph	SVM	76.67	75.04	81.85	87.33

**Table 8:** LOSOCV: F1-score of the proposed algorithm vs other methods.

Papers	Features	Classifier	F1-Score			
			SMIC	CASMEII	CAS(ME)${}^{2}$	SAMM
[13]	Bi-WOOF	SVM	0.62	0.61	0.47	-
[15]	OFF-ApexNet	CNN	0.67	0.87	-	0.54
[24]	LBP-TOP	SMO	-	0.51	-	0.39
	HOOF	SMO	-	0.60	-	0.48
	HOG3D	SMO	-	0.51	-	0.44
[29]	STRCN-A	deep-RCN	0.51	0.54	-	0.49
	STRCN-G	deep-RCN	0.70	0.75	-	0.74
[40]	Facial Dynamics Map	SVM	0.53	0.41	-	-
[41]	STCLQP	SVM	0.64	0.58	-	-
[42]	Bi-WOOF + Phase	SVM	0.67	0.65	-	-
[43]	Hierarchical STLBP-IP	KGSL	0.52	0.32	-	-
Proposed	Full-face graph	SVM	0.65	0.70	0.72	0.80
	FACS-based graph	SVM	0.75	0.74	0.80	0.87

**Table 9:** Computational Performance for the Proposed and Conventional Methods.

Papers	Technique	Resolution	Time	Pre-Processing
[7]	fast LBP-TOP using tensor unfolding	50 × 50	107.39 ms	Required face detection, face alignment, face Cropping and onset-offset detection.
[8]	GPU based LBP-TOP with CUDA	140 × 170	2.98 ms	Required face detection, face cropping, face alignment and onset-offset detection.
[44]	Absolute Two-frame Differences	640 × 480	9 ms	Required face detection and onset-offset detection.
Proposed	Full-face graph with 68-point landmarks	640 × 480	3.1 ms	68-point facial landmarks detection.
Proposed	FACS-based graph with 68-point landmarks	640 × 480	2 ms	68-point facial landmarks detection.

## Abbreviations

The following abbreviations are used in this manuscript:

ME	Micro-expression
FACS	Facial Action Coding System
AU	Action unit
LBP-TOP	Local Binary Pattern histograms from Three Orthogonal Planes
HOG	Histogram of Oriented Gradients
HOOF	Histograms of Oriented Optical Flow
GPU	Graphics processing unit
CUDA	Compute unified device architecture
SMIC	Spontaneous micro-expression
CASMEII	Chinese academy of sciences micro-expression
CAS(ME)${}^{2}$	Spontaneous macro-expressions and micro-expressions
SAMM	Spontaneous actions and micro-movements
